# Meta-Analysis on Dietary Patterns and Pancreatic Cancer Risk: Methodological Limitations

**DOI:** 10.3390/nu9070721

**Published:** 2017-07-07

**Authors:** Shahab Alizadeh, Ahmad Esmaillzadeh

**Affiliations:** 1 Department of Cellular and Molecular Nutrition, School of Nutritional Sciences and Dietetics, Tehran University of Medical Sciences, Tehran P.O. Box 14155-6446, Iran; alizadeh.mnutr@gmail.com; 2 Obesity and Eating Habits Research Center, Endocrinology and Metabolism Molecular Cellular Sciences Institute, Tehran P.O. Box 14155-6117, Iran; 3 Department of Community Nutrition, School of Nutritional Sciences and Dietetics, Tehran University of Medical Sciences, Tehran P.O. Box 14155-6117, Iran

Dear Editor,

We read with great interest the article by Lu et al. [1] about the meta-analysis of 32 studies examining the association between dietary patterns and risk of pancreatic cancer (PC). Despite providing interesting data, the paper has a significant limitation in its identification of dietary patterns; the authors have considered single food items or individual food groups as major dietary patterns, which is not methodologically correct.

The relation of single nutrients or food items with PC has been broadly studied. Since foods and nutrients are never eaten in isolation and their effects are likely to be interacted, a major limitation of the individual-nutrient or food-item approach is their inter-correlations. To overcome this, the dietary patterns approach emerged, in which a more comprehensive understanding of the entire diet and its influence on the etiology of disease could be reached [2]. There are about 45 studies examining the association of single food items and risk of PC, while, to the best of our knowledge, there are only seven studies [3,4,5,6,7,8,9] exploring the association of dietary patterns—either by a priori or a posteriori methods—with PC. In the meta-analysis by Lu et al. [1], single food items or food groups have been considered as dietary patterns. For instance, they considered meat intake in the studies of Taunk et al. [10], Nöthlings et al. [11], and Anderson et al. [12] as a western/unhealthy dietary pattern and pooled the risk estimates of these studies with the results of studies by Nkondjock et al. [5] and Michaud et al. [6], which identified a western/unhealthy pattern (characterized by a higher intake of red meats, sweets, desserts, soft drinks, high-fat dairy products, refined grains, fast foods, and lower loading of fruits and vegetables) through an a posteriori method. It is clear that meat is only one of the components of a western/unhealthy pattern and should be considered as a food item, not as a western/unhealthy dietary pattern. The same limitation exists for the healthy dietary pattern in their meta-analysis. Accordingly, we believe that the results of the study by Lu et al. [1] should be interpreted with caution because of an inappropriate methodology.

In summary, although conducting a meta-analysis is a good approach for summarizing the findings from earlier studies, investigators should take great care when extracting data from previous publications.
